# Retrospective review of patients with chronic lymphocytic leukemia treated with Mohs surgery for nonmelanoma skin cancer

**DOI:** 10.1016/j.jdin.2023.08.006

**Published:** 2023-08-19

**Authors:** Annika P. Weinhammer, Kristen L. Chen, Yaohui G. Xu

**Affiliations:** Department of Dermatology, University of Wisconsin, Madison, Wisconsin

**Keywords:** basal cell carcinoma, chronic lymphocytic leukemia (CLL), keratinocyte carcinoma, Mohs surgery, nonmelanoma skin cancer (NMSC), peritumoral infiltrate, squamous cell carcinoma

*To the Editor:* There is a higher incidence of nonmelanoma skin cancer in patients with chronic lymphocytic leukemia and it seems to exhibit a heightened aggressive nature and higher rates of recurrence after Mohs surgery.^1^^,^^2^ There are many postulated reasons with no well-defined pathogenesis.^3^ Interpretation of histological section can often be complicated by the presence of dense, monomorphic lymphocytic infiltrates as it may not be clear whether the infiltrate is part of the leukemic process or part of a reaction to residual carcinoma.^4^

We performed an institutional review board–approved retrospective review of all patients with chronic lymphocytic leukemia treated with Mohs surgery at the University of Wisconsin Madison within the past 20 years and propose to review the following: (1) demographics, (2) tumor characteristics and outcomes, (3) frequency and patterns of peritumoral infiltrate (defined as dense collections of mononuclear cell aggregates of approximately 50 cells or more) on Mohs slides, and (4) correlation with disease severity at the time of Mohs surgery. For each patient selected, we reviewed all Mohs slides of tumors treated within the past 10 years.

Twenty patients (6 females, 14 males) and 119 tumors were included. Mohs slides were reviewed for the 82 tumors treated in the past 10 years. The tumors included 64 squamous cell carcinomas (SCCs), 38 basal cell carcinomas, 16 squamous cell carcinomas in situ, and 1 “carcinoma.” Brigham Women Hospital staging (for SCCs) included 35 T1 tumors (54.7%), 19 T2a tumors (29.7%), 8 T2b tumors (12.5%), and 2 T3 tumors (3.1%). Five cases had perineural invasion. Peritumoral infiltrate was present on 12/82 (14.6%) tumors in 6 patients. Peritumoral infiltrate was present on every tumor in these 6 patients. The remaining 14 patients had no peritumoral infiltrate present on any of their tumors. Peritumoral infiltrate was present diffusely with focal evidence of residual tumor in 9/12 (75%) of tumors (Fig 1) versus diffusely without clear evidence of residual tumor in 3/12 (25%) of tumors. An additional layer was not taken in the 3 cases without clear evidence of residual tumor. There was no clear correlation with tumor type, stage of SCC, lymph node involvement, systemic treatment, lymphocyte count, or Rai score, which is a prognostic indicator used in chronic lymphocytic leukemia (Table I).^5^ The average number of layers taken on tumors with peritumoral infiltrate present was 2.7 in comparison to 1.9 when no peritumoral infiltrate was present. There were 7 recurrent tumors (5.9%) with no clear correlation to the presence of peritumoral infiltrate. There were no patients with known nodal or metastatic disease.

**Fig 1:**
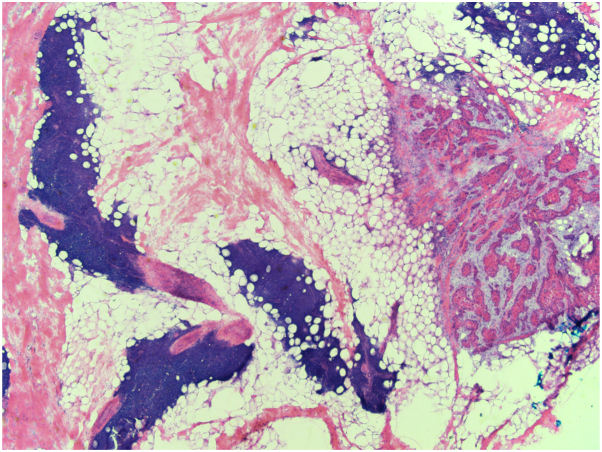
Mohs slide from tumor with peritumoral infiltrate and focal evidence of residual tumor present (40×).

**Table I:** Characteristics of tumors with peritumoral infiltrate

Patient	Age	Tumor type	Location	SCC BWH staging	# of Mohs layers	Peritumoral infiltrate pattern	Rai score∗	Lymphocyte count	Node involvement	Systemic treatment
1	74	BCC	L preauricular cheek	x	1	Diffuse with tumor present	1	20	Yes	None
1	74	BCC	L nasal sidewall	x	3	Diffuse with tumor present	1	20	Yes	None
2	79	SCC	Vertex scalp	T3	3	Diffuse with tumor present	0	1230	No	None
2	80	BCC	R nasal sidewall	x	2	Diffuse with tumor present	0	1230	No	None
3	83	SCC	L cheek	T1	2	Diffuse with tumor present	1	17,914	Yes	None
3	84	BCC	L upper lip	x	3	Diffuse with tumor present	4	79,442	Yes	None
3	86	SCC	R temple	T2a	1	Diffuse without tumor present	3	248,560	No	None
3	87	SCC	Vertex scalp	T2a	2	Diffuse with tumor present	3	17,990	No	Ibrutinib
4	85	SCC	L lateral forehead	T3	11	Diffuse with tumor present	1	17,384	No	None
5	76	BCC	L nasal ala	x	1	Diffuse without tumor present	x	Unknown	Unknown	Unknown
6	79	SCC	Midline chest	T1	1	Diffuse without tumor present	4	16,800	No	None
6	79	SCC	R upper arm	T2a	2	Diffuse with tumor present	4	16,800	No	None

*BCC*, Basal cell carcinoma; *BWH*, Brigham Women Hospital; *SCC*, squamous cell carcinoma.

∗ Rai score is a prognostic indicator used in patients with chronic lymphocytic leukemia with lymphocytosis. It is calculated based on 5 categories including enlarged lymph nodes, enlarged spleen, enlarged liver, anemia (Hgb <11 g/dL), and thrombocytopenia (platelets <100,000/mm^3^)

There was a higher SCC:basal cell carcinoma ratio (1.7:1) in comparison to general population. 10/64 (15.6%) of SCCs were “high risk” (Brigham Women Hospital T2b or higher). Peritumoral infiltrate was present on 12/82 (14.6%) of tumors with majority having some residual tumor present (75%). Peritumoral infiltrate appeared to be unique to individual patients which suggests that the infiltrate is attributable to patient or disease specific factors rather than an association with the tumor itself. Further studies to support this conclusion include a prospective study design to collect clinical, immunologic, and genetic data.

## Conflicts of interest

None disclosed.
